# Effects of Adper^™^ Scotchbond^™^ 1 XT, Clearfil^™^ SE Bond 2 and Scotchbond^™^ Universal in Odontoblasts

**DOI:** 10.3390/ma14216435

**Published:** 2021-10-27

**Authors:** Miguel Cardoso, Ana Coelho, Carlos Miguel Marto, Ana Cristina Gonçalves, Anabela Paula, Ana Bela Sarmento Ribeiro, Manuel Marques Ferreira, Maria Filomena Botelho, Mafalda Laranjo, Eunice Carrilho

**Affiliations:** 1 Institute of Integrated Clinical Practice, Faculty of Medicine, University of Coimbra, 3000-075 Coimbra, Portugal; anasofiacoelho@gmail.com (A.C.); cmiguel.marto@uc.pt (C.M.M.); anabelabppaula@sapo.pt (A.P.); eunicecarrilho@gmail.com (E.C.); 2 Institute of Biophysics, Faculty of Medicine, University of Coimbra, 3000-548 Coimbra, Portugal; mfbotelho@fmed.uc.pt (M.F.B.); mafaldalaranjo@gmail.com (M.L.); 3 Institute for Clinical and Biomedical Research (iCBR), Area of Environment, Genetics and Oncobiology (CIMAGO), Faculty of Medicine, University of Coimbra, 3000-548 Coimbra, Portugal; acc.goncalves@gmail.com (A.C.G.); absarmento@fmed.uc.pt (A.B.S.R.); mmferreira@fmed.uc.pt (M.M.F.); 4 Center for Innovative Biomedicine and Biotechnology (CIBB), University of Coimbra, 3000-548 Coimbra, Portugal; 5 Clinical Academic Center of Coimbra (CACC), 3004-561 Coimbra, Portugal; 6 Institute of Experimental Pathology, Faculty of Medicine, University of Coimbra, 3000-548 Coimbra, Portugal; 7 Laboratory of Oncobiology and Hematology (LOH), Faculty of Medicine, University of Coimbra, 3000-548 Coimbra, Portugal; 8 Institute of Endodontics, Faculty of Medicine, University of Coimbra, 3000-075 Coimbra, Portugal

**Keywords:** dental adhesives, adhesive systems, cytotoxicity, Odontoblasts, cell culture

## Abstract

This study aimed to assess the cytotoxicity of commercially available adhesive strategies—etch-and-rinse (Adper^™^ Scotchbond^™^ 1 XT, 3M ESPE, St. Paul, MN, USA, SB1), self-etch (Clearfil^™^ SE Bond 2, Kuraray Noritake Dental Inc., Tokyo, Japan, CSE), and universal (Scotchbond^™^ Universal, 3M Deutschland GmbH, Neuss, Germany, SBU). MDPC-23 cells were exposed to adhesives extracts in different concentrations and exposure times. To access cell metabolic activity, viability, types of cell death, and cell cycle, the MTT assay, SRB assay, double labeling with annexin V and propidium iodide, and labeling with propidium iodide/RNAse were performed, respectively. Cultures were stained with May-Grünwald Giemsa for qualitative cytotoxicity assessment. The SB1, CSE, and SBU extracts determined a significant reduction in cell metabolism and viability. This reduction was higher for prolonged exposures, even for less concentrated extracts. CSE extracts significantly reduced the cell’s metabolic activity at higher concentrations (50% and 100%) from 2 h of exposure. After 24 and 96 h, a metabolic activity reduction was verified for all adhesives, even at lower concentrations. These changes were dependent on the adhesive, its concentration, and the incubation time. Regarding cell viability, SBU extracts were the least cytotoxic, and CSE was significantly more cytotoxic than SB1 and SBU. The adhesives determined a reduction in viable cells and an increase in apoptotic, late apoptosis/necrosis, and necrotic cells. Moreover, on cultures exposed to SB1 and CSE extracts, a decrease in the cells in S and G2/M phases and an increase in the cells in G0/G1 phase was observed. Exposure to SBU led to an increase of cells in the S phase. In general, all adhesives determined cytotoxicity. CSE extracts were the most cytotoxic and were classified as having a higher degree of reactivity, leading to more significant inhibition of cell growth and destruction of the cell’s layers.

## 1. Introduction

In modern dentistry, adhesive systems allow the adhesion of restorative materials to the dental substrate. This is fundamental for the recovery of dental esthetics and function after tissue loss by dental caries or fractures or to perform color changes and improve teeth positioning and shape [1,2].

Dental adhesives can be classified according to their clinical application as etch-and-rinse, self-etch, and multi-mode/universal. The etch-and-rinse adhesives require the etching of the tooth structure with phosphoric acid before applying the primer and the bond solution. By incorporating acidic monomers, the self-etch adhesives have the intrinsic capacity of etching the surface without prior acid application. This adhesive strategy incorporates the smear layer created after the mechanical tooth preparation in the adhesive interface, which leads to the preservation of the dentin collagen fibers. At last, the universal systems allow the application over any substrate, acid-etched or not [1,3,4]. Adhesion protocols may be adapted according to the dental substrate present. This way, when only enamel is present, all adhesive strategies are appropriate options if the enamel is previously etched with phosphoric acid. When adhering to dentin, adhesives that integrate the smear layer (self-etch or universal) may be used. In cavities with both enamel and dentin, self-etch or universal systems can be used with selective acid-etching of the enamel. The recommended adhesion protocols and their variations according to the adhesive system presentation are presented in Table 1 [1,3].

Like most biomaterials for medical applications, adhesives systems producers keep launching new and improved materials into the market [5,6]. This evolution intends to simplify their clinical application and enhance their performance, increasing the longevity of the restorations [7,8,9]. To achieve this, adhesives present complex chemical formulations, and several substances in these materials can induce adverse biological reactions [4,10]. Triethylene glycol dimethacrylate (TEGDMA) and 2-hydroxyethyl methacrylate (HEMA) are frequent monomers used in adhesive formulations with related cytotoxicity [4,11].

The dentin acid-etching amplifies the interaction of the adhesive system with the dentin-pulpal complex once it results in the exposure of the dentinal tubules, facilitating the adhesive diffusion [12,13]. Additionally, incomplete polymerization may increase the toxicity of the adhesive system by a higher concentration of free compounds or the diffusion of substances through the dentinal tubules [12,14].

The adhesives which do not require the dentin acid-etching incorporate the smear layer in the adhesive interface [2,3,15]. Given the non-exposure of the dentinal tubules, these systems penetrate less into the dentin. This way, there is a lower probability of adverse reactions in the dentin-pulpal complex compared with adhesion over acid-etched dentin [16,17].

In deep dentin cavities and independently of the adhesive strategy, the adhesive toxic compounds may easier diffuse through the dentinal tubules and reach the pulp [12,16]. Indirect pulp capping can reduce the adhesive reaching the pulp; however, it reduces the total adhesion area, which mechanically compromises the adhesion [18,19,20].

Although with some limitations, several studies have evaluated adhesive systems cytotoxicity [6,16,21,22,23]. Frequently, adhesives from one strategy only [23,24,25] are used, and the cellular response characterization is incomplete, relying on few techniques. Additionally, most of the studies have used fibroblasts [26,27,28] or extra-oral cells [29] and followed critically different protocols. Thus, a robust and comprehensive comparison of the cytotoxicity of the three adhesive strategies based on reproducible and biologically relevant experimental models is lacking. The present study aimed to access the in vitro cytotoxicity of adhesive systems representative of the three commercially available strategies—etch-and-rinse, self-etch, and universal.

## 2. Materials and Methods

For all procedures, the cell cultures were treated according to the ISO 10993-5 (International Standard of Biological Evaluation of Medical Devices—part 5: tests for in vitro cytotoxicity) recommendations [30], which establish the adequate procedures for the in vitro cytotoxicity assessment of medical devices.

### 2.1. Adhesive Systems

Three adhesives were tested: Adper™ Scotchbond™ 1 XT (SB1, 3M ESPE, St. Paul, MN, USA), Clearfil™ SE Bond 2 (CSE, Kuraray Noritake Dental Inc., Tokyo, Japan), and Scotchbond™ Universal (SBU, 3M Deutschland GmbH, Neuss, Germany). The adhesive strategy, batch number, and material presentation are detailed in Table 2.

All cell culture procedures were performed in a laminar flow chamber (Holten LaminAir HBB 2448, Holten, Denmark), according to the aseptic technique for keeping the sterility of the materials, supplements, and culture medium [31,32].

The adhesives were placed into polyvinyl chloride (PVC) molds with 4 mm diameter, in a total volume of 10 µL. For Clearfil™ SE Bond 2, the two bottles were added in equal parts, and pellets were obtained from a volume of 10 µL. The adhesives were polymerized for 20 s with a Bluephase^®^ Style curing unit (Ivoclar Vivadent, Schaan, Liechtenstein) until a solid substrate was obtained (pellets).

After polymerization, the pellets were incubated with Dulbecco’s Modified Eagle’s Medium (DMEM, 13.4 g/L—D-5648, Sigma Aldrich, St. Louis, MO, USA), pH 7.4, supplemented with 10% fetal bovine serum (FBS, F7524, Sigma Aldrich, St. Louis, MO, USA), 3.7 g/L of sodium bicarbonate (S-5761, Sigma Aldrich, St. Louis, MO, USA), 250 μM of sodium pyruvate (11360 Gibco™, Thermo Fisher Scientific, Austin, TX, USA), and 1% of antibiotic and antifungal solution (10,000 U/mL penicillin, 10 mg/mL streptomycin, and 25 μg/mL amphotericin B, A5955, Sigma Aldrich, St. Louis, MO, USA). Following published protocols [23,33], the conditioned media, named extracts, were prepared with a relation of one pellet per mL of medium, and kept for 24 h in falcon tubes (Sarstedt 62.554.502, Nümbrecht, Germany) under stirring, in a HeraCell^®^ 150 incubator (Thermo Electron Corporation, Palm Beach, FL, USA), with 95% relative humidity, 5% of CO_2_, and temperature of 37 °C. After incubation, the extracts were centrifuged for 5 min at 1600× *g*. Then, the supernatant was collected, and the intended dilutions were performed with fresh DMEM.

The metabolic activity assays evaluated the extracts at the concentrations of 6.25%, 12.5%, 25%, 50%, and 100%. The remaining tests evaluated the concentrations of 25% and 50%. Controls were established for all the assays, corresponding to cultures exposed to fresh DMEM.

### 2.2. Cell Cultures

In the present work, a mouse dental papilla odontoblast-like cell line (MDPC-23) was used. This cell line was gently given by Professor Jacques E. Nör from the University of Michigan, Ann Arbor, MI, USA. This cell line is recommended for the in vitro assessment of dental materials biocompatibility [33,34]. Cells were defrosted and propagated in adherent conditions in 75 cm^2^ flasks (Sarstedt 83.3911.002, Nümbrecht, Germany) and maintained in the incubator during all study phases. The cells were detached using TrypLE™ Express Enzyme (12605028, Gibco™, Thermo Fisher Scientific, Austin, TX, USA), counted using trypan blue 0.4% (T8154, Sigma Aldrich, St. Louis, MO, USA), and distributed in cell culture plates.

### 2.3. Cytotoxicity Assessment

Suspensions of 50.000 cells/mL were distributed into 96-well culture plates (Sarstedt 83.3924, Nümbrecht, Germany) for the 3-(4,5-dimethylthiazol-2-yl)-2,5-diphenyltetrazolium bromide (MTT) assay, or into 48-well culture plates (Sarstedt 83.3923, Nümbrecht, Germany) for the sulforhodamine B (SRB) assay. After plating, cell cultures were incubated for 24 h to allow the adherence of the cells.

Then, the culture medium was replaced by an equal volume of the adhesive extracts at the different concentrations of interest. The surrounding wells were filled with fresh DMEM to ensure equal humidity conditions between wells. The cell cultures were incubated for 2, 24, and 96 h for the MTT assay and 24 h for the SRB assay.

#### 2.3.1. Metabolic Activity

To evaluate the metabolic activity, the MTT assay was performed. First, the medium was removed, and the wells were washed with PBS. The MTT solution (0.5 mg/mL in PBS, pH 7.4; M2128, Sigma Aldrich, St. Louis, MO, USA) was added, and the plates were incubated away from light, at 37 °C, overnight. A solution of 0.04 M of hydrochloric acid (84415, Sigma Aldrich, St. Louis, MO, USA) in isopropanol (563935, Sigma Aldrich, St. Louis, MO, USA) was added to dilute the formazan crystals, and the plates were stirred for 45 min. The absorbance was quantified using the spectrophotometer EnSpire^®^ (PerkinElmer Inc., Shelton, CT, USA) with a wavelength of 570 nm and a reference filter at 620 nm. The results are presented as the percentage of the metabolic activity of the test cell cultures in relation to the corresponding control cultures.

#### 2.3.2. Cell Viability

To evaluate cell viability, the SRB assay was performed. After 24 h of incubation with the extracts, the medium was removed, and the wells were washed with PBS. Cells were fixed with acetic acid (ARK2183, Sigma Aldrich, St. Louis, MO, USA) at 1% in methanol (322415, Sigma Aldrich, St. Louis, MO, USA) for 1 h at 4 °C. After drying, a solution of SRB (S9012, Sigma Aldrich, St. Louis, MO, USA) at 4% in acetic acid at 1% was added, and the plates were dyed for 2 h, at room temperature, in the dark. Finally, the wells were washed, dried, and Tris.NaOH (10 mM; T1503, Sigma Aldrich, St. Louis, MO, USA) was added.

The absorbance was quantified in the spectrophotometer EnSpire^®^ with a wavelength of 570 nm and a reference filter at 690 nm. The results are presented as the percentage of the test cell cultures in relation to the corresponding control cultures.

### 2.4. Flow Cytometry Studies

The cells were seeded in 6-well plates (Sarstedt 83.3920, Germany) at 500.000 cells/well, left for 24 h for adherence, and then the extracts were added. After 24 h of incubation, the wells were washed with PBS, and the cells detached and centrifuged at 1000× *g* for 5 min.

#### 2.4.1. Types of Cell Death

Types of cell death were determined by flow cytometry using double labeling with annexin V (AnV) fluorescein isothiocyanate (FITC) and propidium iodide (PI). Cells were labeled in binding buffer (constituted by 0.01 M of Hepes (H7523, Sigma Aldrich, St. Louis, MO, USA), 0.14 mM of NaCl (S7653, Sigma Aldrich, St. Louis, MO, USA) and 0.25 mM of CaCl2 (C4901, Sigma Aldrich, St. Louis, MO, USA)), with AnV FITC and IP as recommended by the kit supplier (KIT Immunotech, Marseille, France). The suspensions were homogenized and analyzed in the cytometer FACSCalibur (BD Biosciences, Becton Dickinson, Franklin Lakes, NJ, USA). The software Paint-a-Gate™ 3.02, Macintosh Software (BD Biosciences, Becton Dickinson, Franklin Lakes, NJ, USA) was used for the analysis and quantification. The results show the percentage of living, apoptotic, late apoptotic/necrotic, and necrotic cells present in the cultures.

#### 2.4.2. Cell Cycle

The cell cycle was assessed by flow cytometry through the labeling of the cells with PI. After centrifugation, the cells were fixed with 70% ethanol for 30 min at 4 °C in the dark. After washing, the PI/RNase solution (Immunotech, Marseille, France) was added. After incubation, the cell suspensions were analyzed in the FACSCalibur cytometer at the excitation wavelength of 488 nm and 582 nm of emission. The quantification was performed using the software ModFit LT™ (Verity Software House, Topsham, ME, USA). The results are presented by the percentage of cells in the phases Pre-G0, G0/G1, S, or G2/M present in the cultures.

### 2.5. Morphology and Qualitative Cytotoxicity Assessment

Cell morphology was assessed by optical microscopy through the staining of the cells with May-Grünwald Giemsa.

Cells were seeded in 12-well plates (Sarstedt 83.3921, Nümbrecht, Germany) over a round sterile coverslip. After cell adhesion, the extracts were added, and the medium of the control wells was replaced with fresh DMEM. The plates were incubated for 24 h. The supernatant was decanted, and the wells were washed with PBS. Next, the May-Grunwald solution (Laborclin, Pinhais, Paraná, Brazil) was added and, after 3 min of incubation, an equal volume of ultrapure water was added. The supernatant was decanted, and the Giemsa solution (Laborclin, Brazil) was added. Then, the coverslips were washed with tap water and mounted in microscopic slides using DAKO glycergel (DAKO, Glostrup, Denmark). The cells were analyzed in an optical microscope and photographed using a Nikon OS-Fi2 (Nikon, Tokyo, Japan) camera at 100× magnification.

The qualitative assessment of cytotoxicity was performed based on analyzing six random photographs for each experimental condition. The grading of reactivity is described in the ISO 10993-5 [30]. Briefly, no reactivity is considered when no cell lysis and no reduction of cell growth is observed; slight reactivity corresponds to up to 20% round cells, loosely attached, without intracytoplasmic granules or other morphological changes and slight growth inhibition; mild reactivity is attributed to up to 50% round cells, loosely attached, without intracytoplasmic granules or other morphological changes and up to 50% growth inhibition; moderate reactivity corresponds to up to 70% round cells, loosely attached, without intracytoplasmic granules or other morphological changes and more than 50% growth inhibition; and severe reactivity corresponds to complete or nearly complete destruction of the cell layers.

### 2.6. Statistical Analysis

The statistical analysis was performed using the software GraphPad Prism 8^®^ (San Diego, CA, USA). For all comparisons, a 5% significance value was considered. The Shapiro-Wilk test was used to assess the normal distribution of all quantitative variables.

For the metabolic activity and cell viability evaluation, the cells submitted to the extracts were compared to the control cultures (normalized at 100%), using the t-student test for one sample, when normal distribution was verified. Otherwise, the Wilcoxon test was used. The experimental conditions were compared along the incubation periods using the one or two-factor ANOVA, as applicable when normal distribution was present. Otherwise, the Kruskal-Wallis test was performed. Multiple comparisons and respective correction were performed, using the Tukey or Dunn corrections for parametric and non-parametric tests, respectively.

Regarding the types of cell death and cell cycle analysis, comparisons between the test and control cultures were performed using the one-factor ANOVA when normal distribution was present. Otherwise, the Kruskal-Wallis test was used. Multiple comparisons were made, and the corrections of Bonferroni or Dunn were used as applicable.

Results are presented as the mean and standard error of the mean.

## 3. Results

### 3.1. Metabolic Activity

**Figure 1:**
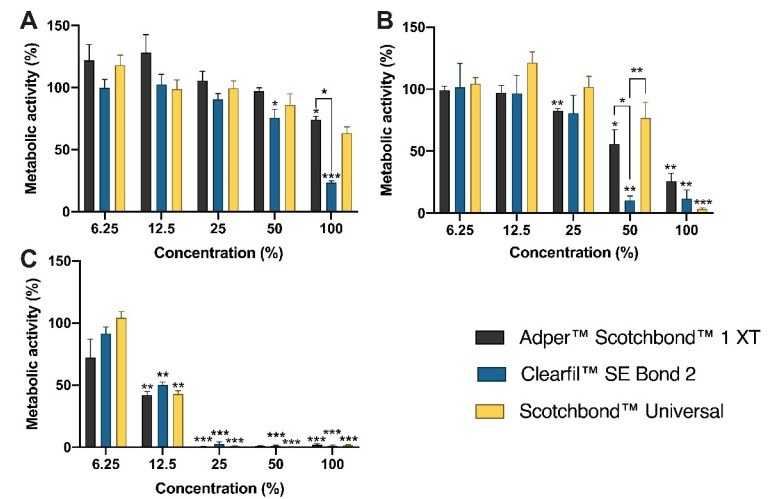
Metabolic activity of MDPC-23 cells after incubation with the adhesive extracts for 2 h (**A**), 24h (**B**), and 96 h (**C**). Results are presented as the mean and standard error of the mean of four independent experiments. Statistically significant differences are presented with *, where * means *p* < 0.05, ** means *p* < 0.01, and *** means *p* < 0.001.

The results from the metabolic activity of the MDPC-23 cells, obtained with the MTT assay, are presented in Figure 1.

After 2 h of incubation, the extracts of SB1 determined a significant decrease of the metabolic activity to 74.17 ± 2.63% (*p* = 0.010) after exposure to the concentration 100%. The exposure to CSE at 50% and 100% also led to a significant reduction of the metabolic activity to 75.77 ± 6.86% (*p* = 0.039) and 23.49 ± 1.41% (*p* < 0.001), respectively. At the 100% concentration, a more significant metabolic activity reduction was seen after exposure to CSE compared with SB1 (*p* = 0.034).

Incubating the cultures for 24 h with SB1 extracts significantly decreased the metabolic activity to 82.64 ± 1.65% (*p* = 0.009), to 33.55 ± 13.79% (*p* = 0.041), and to 25.84 ± 6.40% (*p* = 0.007), at the concentrations of 25%, 50%, and 100%, respectively. The extracts of CSE at 50% and 100% significantly reduced the metabolic activity to 10.40 ± 3.36% (*p* = 0.001) and to 11.60 ± 7.22% (*p* = 0.001), respectively. The exposure to SBU at 100% concentration determined the significant reduction of the metabolic activity to 3.33 ± 1.17% (*p* < 0.001). At 50% concentration, the extracts of CSE significantly reduced the metabolic activity compared with SB1 (*p* = 0.044) and SBU (*p* = 0.008).

After 96 h of incubation, the SB1 extracts significantly reduced the metabolic activity to 41.98 ± 2.88% (*p* = 0.003), 0.85 ± 0.04% (*p* < 0.001), and 2.03 ± 0.97% (*p* < 0.001), at 12.5%, 25%, and 100% concentrations, respectively. CSE extracts determined a significant reduction of the metabolic activity to 49.88 ± 2.56% (*p* = 0.003), 2.61 ± 1.75% (*p* < 0.001), 1.22 ± 0.50% (*p* < 0.001), and 1.27 ± 0.55% (*p* < 0.001), at 12.5%, 25%, 50%, and 100% concentrations, respectively. Finally, the SBU extracts determined a significant reduction of the metabolic activity to 42.87 ± 2.53% (*p* = 0.002), to 1.14 ± 0.30% (*p* < 0.001), to 0.61 ± 0.13% (*p* < 0.001), and to 1.49 ± 0.55% (*p* < 0.001), after exposure to 12.5%, 25%, 50%, and 100% concentrations, respectively.

Except for SBU at 6.25% concentration, all adhesives at all concentrations (determined a significant decrease in the metabolic activity leading it to residual values after 96 h compared with 2 h of incubation.

### 3.2. Cell Viability

**Figure 2:**
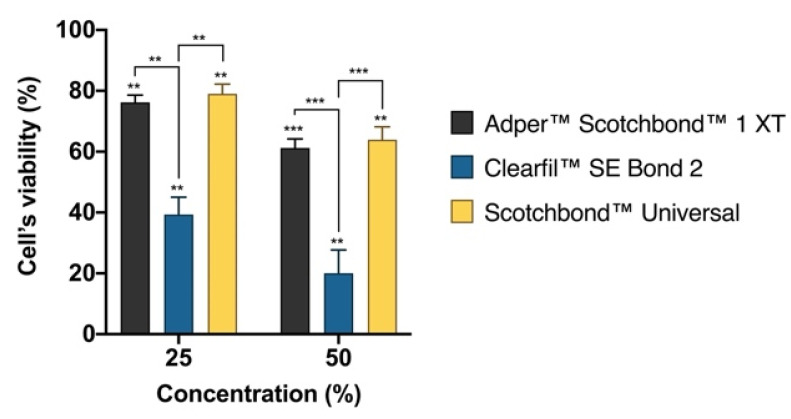
Protein content of MDPC-23 cells when submitted to the adhesive extracts for 24 h. Results are presented as the mean and standard error of the mean of three independent experiments. Statistically significant differences are presented with *, where ** means *p* < 0.01, and *** means *p* < 0.001.

The SRB assay results regarding the cell viability of the MDPC-23 cultures after incubation for 24 h are presented in Figure 2.

The SB1, CSE, and SBU extracts significantly reduced cell viability at both 25% and 50% concentration, after 24 h of incubation. Thus, cell viability decreased to 76.22 ± 2.34% (*p* = 0.002) after exposure to SB1 at 25% concentration, and to 61.18 ± 2.98% (*p* = 0.001) at 50% concentration. CSE extracts determined the highest decrease in cell viability to 39.33 ± 5.69% (*p* = 0.009) after exposure to 25% concentration, and to 20.01 ± 7.69% (*p* = 0.009) after 50% concentration. The extracts of SBU significantly reduced cell viability to 79.01 ± 3.22% (*p* = 0.007) after exposure to 25% concentration, and to 63.90 ± 4.26% (*p* = 0.003) after 50% concentration.

At both tested concentrations, CSE extracts determined a significant reduction of cell viability compared with SB1 (*p* = 0.002) and SBU (*p* = 0.001), at 25% and SB1 (*p* < 0.001) and SBU (*p* < 0.001) at 50% concentration.

### 3.3. Types of Cell Death

**Figure 3:**
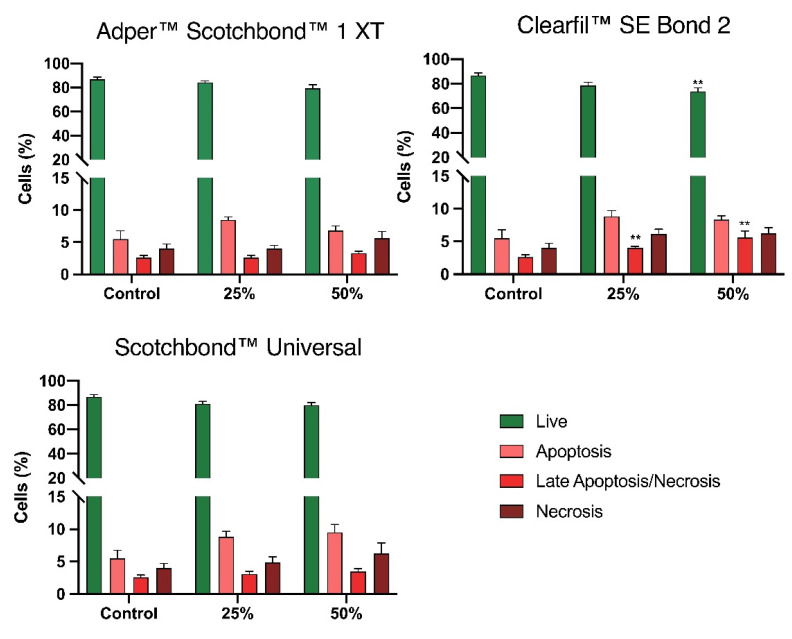
Cell viability was determined by flow cytometry using the annexin-V/propidium iodide (AV/PI) incorporation assay. Results are presented as the mean and standard error of the mean of five independent experiments. Statistically significant differences are presented with *, where ** means *p* < 0.01.

Results concerning the types of cell death are shown in Figure 3. The control cultures had a mean percentage of 86.8 ± 1.9% live cells, 7.0 ± 1.4% apoptotic cells, 2.3 ± 0.3% late apoptotic/necrotic cells, and 4.0 ± 0.7% necrotic cells.

After exposure to SB1 at 25% concentration, there was a slight decrease in the percentage of live cells to 84.3 ± 1.4%. This decrease was accompanied by an increase in the percentage of apoptotic cells to 9.6 ± 1.1%. For 50% concentration, there was a decrease in live cells to 79.4 ± 3.0%, and an increase of cells in apoptosis to 12.0 ± 3.4%, in late apoptosis/necrosis to 3.0 ± 0.3%, and in necrosis to 5.6 ± 1.0%. The exposure to SBU extracts at 25% concentration determined the decrease of live cells 81.1 ± 2.1%, with an increase of cells in apoptosis to 11.2 ± 2.0%, in late apoptosis/necrosis to 3.0 ± 0.5%, and in necrosis to 4.9 ± 0.9%. For 50% concentration, there was a decrease in live cells to 80.1 ± 2.0%, with an increase of cells in apoptosis 12.5 ± 2.3%, in late apoptosis/necrosis to 3.1 ± 0.4%, and in necrosis of 6.3 ± 1.6%. Despite these trends, no statistical differences were identified between the control cultures and the ones exposed to the SB1 and SBU extracts.

CSE extracts at 25% concentration determined the decrease of live cells to 78.6 ± 2.7%, with an increase of apoptotic cells to 11.6 ± 2.8%, and necrotic to 6.1 ± 0.7%. There was also a significant increase of apoptotic/necrotic cells to 3.7 ± 0.2% (*p* = 0.004). For 50% concentration, there was a significant decrease in live cells to 73.8 ± 2.9% (*p* = 0.001), with an increase of cells in apoptosis to 12.5 ± 2.8% and in necrosis to 6.3 ± 0.8%. Additionally, a significant increase of cells in late apoptosis/necrosis to 5.5 ± 1.2% (*p* = 0.001) was observed.

### 3.4. Cell Cycle

**Figure 4:**
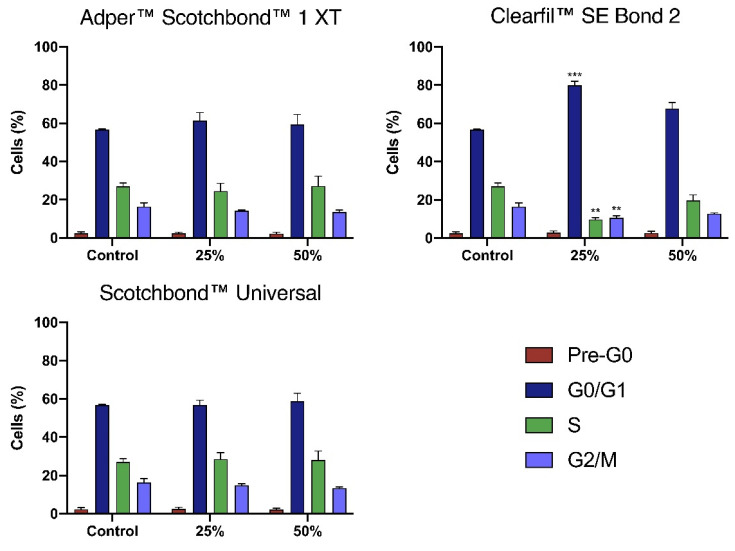
Cell cycle analysis was determined by flow cytometry using the propidium iodide/RNAse incorporation assay. Results are presented as the mean and standard error of the mean of three independent experiments. Statistically significant differences are presented with *, where ** means *p* < 0.01, and *** means *p* < 0.001.

Results from the cell cycle assessment are shown in Figure 4.

The control cultures had a mean percentage of cells in the Pre-G0 phase of 2.3 ± 0.8%, in G0/G1 phase of 56.7 ± 0.3%, in S phase of 27.0 ± 1.8%, and in the G2/M phase of 16.3 ± 2.1%.

The extracts of SB1 at 25% concentration led to an increase of cells in G0/G1 phase to 61.3 ± 4.3%, and a decrease of cells in the S phase to 24.5 ± 4.1%, and in G2/M phase to 14.2 ± 0.5%. At 50% concentration, the extracts determined an increase of cells in G0/G1 phase to 59.3 ± 5.3%, and a decrease of cells in G2/M phase to 13.5 ± 1.1%. SBU extracts at 25% concentration determined a slight increase of cells in the S phase to 28.5 ± 3.4% and a decrease in the G2/M phase to 14.8 ± 0.9%. At 50% concentration, there was a slight increase in the percentage of cells G0/G1 phase to 58.7 ± 4.2%, of cells in the S phase to 28.0 ± 4.8, and a decrease of cells in G2/M phase to 13.3 ± 0.7%. Despite these trends, no statistical differences were identified between the control cultures and the ones exposed to the SB1 and SBU extracts.

The exposure to CSE extracts at 25% concentration increased the percentage of cells in the Pre-G0 phase to 2.7 ± 1.1%, and significantly increased the number of cells in G0/G1 phase to 79.8 ± 2.2% (*p* < 0.001). This increase was accompanied by a significant decrease in the percentage of cells in the S phase to 9.7 ± 1.0% (*p* = 0.009) and in G2/M phase to 10.5 ± 1.2 (*p* = 0.005). At 50% concentration, there was a similar percentage of cells Pre-G0 phase of 2.5 ± 1.0%, an increase of cells in G0/G1 phase to 67.7 ± 3.3%, and a decrease of cells in the S phase to 19.7 ± 2.8%, and in G2/M phase to 12.7 ± 0.6%.

### 3.5. Morphology and Qualitative Cytotoxicity Assessment

Results of the morphology and qualitative cytotoxicity assessment of the MDPC-23 cells are presented in Table 3. These results represent the mean of the grading performed in photographs of control and test cultures in three independent experiments.

**Figure 5:**
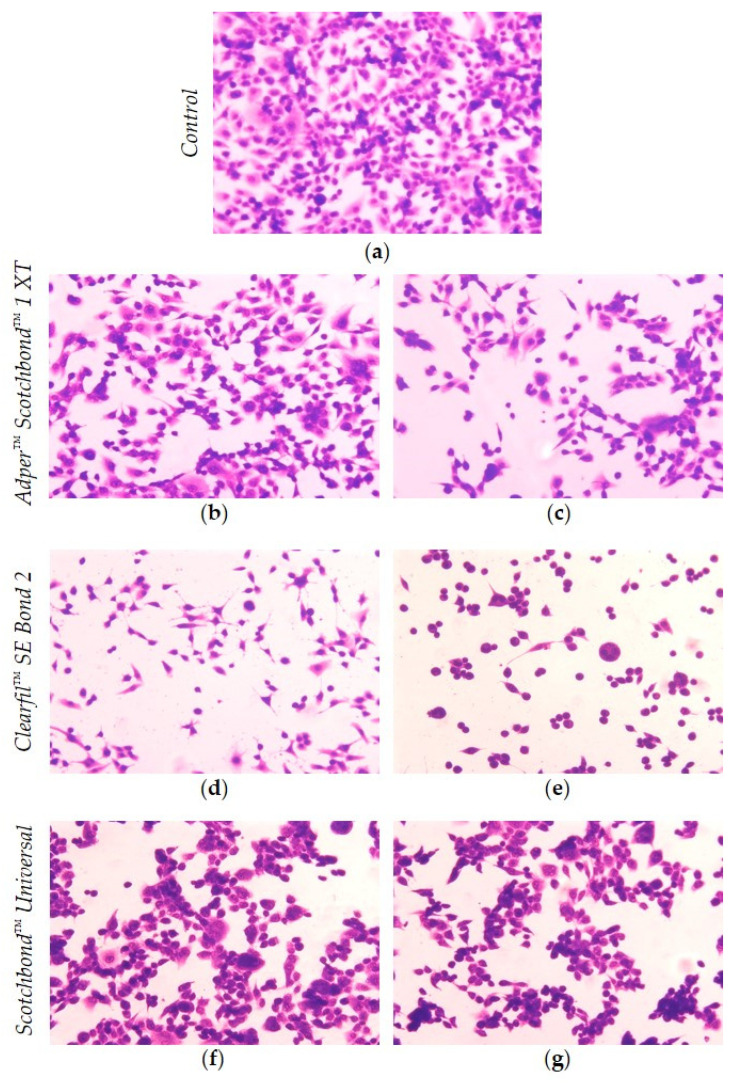
MDPC-23 cells after *May-Grünwald Giemsa* staining. The photographs represent control cultures submitted to DMEM (**a**) and cultures submitted to the extracts of Adper^™^ Scotchbond^™^ 1 XT, at 25% (**b**) and 50% (**c**) concentration, Clearfil^™^ SE Bond 2, at 25% (**d**) and 50% (**e**) concentration, and of Scotchbond^™^ Universal, at 25% (**f**) and 50% (**g**) concentration. These images were obtained by optical microscopy with 100× magnification and are representative of the three independent experiments.

The SB1 extracts at 25% concentration were graded as slight reactive, where a small percentage of round cells was noted, with loss of adherence or morphological alterations. SB1 extracts at 50% concentration and SBU extracts at 25% and 50% concentrations were graded as mild reactive with inhibition of culture growth close to 50%. The CSE extracts at 25% concentration were graded as moderate reactive and at 50% concentration as severe reactive, where morphological alterations and nearly complete destruction of the cultures were noted. Representative photographs are presented in Figure 5.

## 4. Discussion

The studies evaluating adhesive systems cytotoxicity using extracts and direct or indirect contact methodologies showed mixed results and conclusions. For instance, in the study of Koulaouzidou et al. [29], etch-and-rinse adhesives were more cytotoxic than self-etching adhesives. However, other authors reported that self-etch adhesives were the most cytotoxic [21,25,26]. Pagano et al. [23] reported that the universal adhesive studied was the least cytotoxic, but the difference was not statistically significant. Additionally, published papers report that etch-and-rinse and self-etch adhesives determined equal cytotoxicity for the cultures used [28,35]. Caldas et al. [18] explained this heterogenicity in a systematic review on adhesive systems cytotoxicity. Most of the studies had flaws in their methodology, where only four out of ten selected studies were performed following the guidelines of ISO 10993-5. The authors also noted that several methodologies had been used, with different cell lines used and different extraction or exposure protocols, reducing the comparisons’ robustness. Additionally, in the cited studies, cytotoxicity was assessed mainly by differential metabolic activity, considering the control and exposed cultures. Additionally, countless adhesive systems are available in the market, with multiple combinations of components, even between adhesives classified within the same adhesive strategy. This factor alone may be responsible for the previously mentioned variability of results available in the literature. In dentin barrier tests, pH and smear dissolution are not related to cytotoxicity, but the dentin specimen thickness becomes critical. Additionally, there are different reported protocols of sterilization of the specimens, which determines the preservation or destruction of the collagen fibers and, consequently, the capacity of adhesive infiltration into the tubules [27].

This way, a complete overview of the adhesive’s interaction with cells was not fully established, which justifies the need for further research in this topic. Importantly, comparing the three adhesive strategies in the same study can provide relevant information.

To overcome the referred limitations and provide more complete information, experimental procedures should be performed following standardized guidelines, which help compare the obtained results and conclude about the materials cytotoxic risk. This study was developed following the widely accepted ISO 10993-5 guidelines. The extracts methodology was chosen as adhesives are not directly applied over the pulp. However, in deep dentin cavities and other clinical situations, diffusion of the adhesive systems might occur through the dentinal tubules, eventually reaching the pulp [12,16]. When reaching the pulp, the odontoblastic layer is the first affected, so MDPC-23 cells stand as an excellent model to study the cytotoxicity of these materials.

The ISO 10993-5 standard also states that the extracts should not be processed after being obtained. Nevertheless, our initial results showed inconsistency and were hard to reproduce. Thus, it was necessary to centrifugate the extracts before their incubation within the cultures. This procedure led to reproducible results by removing possible pellet fragments or precipitates formed.

The MTT assay results revealed a metabolic activity reduction dependent on the incubation time, the extracts concentrations, and the adhesive type. The Clearfil™ SE Bond 2 extracts determined increased reductions in the metabolic activity compared with the other adhesives studied. After 24 h of incubation, at 50% concentration, there was a significant reduction of the metabolic activity for the exposure to the Clearfil™ SE Bond 2 extracts compared with Adper™ Scotchbond™ 1 XT and Scotchbond™ Universal. Because the DMEM medium changed color instantly when in contact with the Clearfil™ SE Bond 2 pellets, higher cytotoxicity for this material was predicted at the higher concentrations, attributed to a more acidic pH value. It seems to be consensual that when using extracts and direct or indirect contact studies, self-etch adhesives where the primer is separated from the bonding tend to be more cytotoxic, especially related to toxic properties of the primer. This interaction is still dependent on essential factors like the pH of the adhesive and the functional monomers present [36]. The obtained results from the MTT assay determined to explore these experimental conditions in further assays.

Although being frequently used, the MTT assay alone is insufficient to assess material cytotoxicity once decreased metabolic activity does not necessarily mean cell death [37]. We performed cell viability analysis which was reduced after exposure to all adhesives, both at 25% and 50% concentrations, where the extracts of Clearfil™ SE Bond 2 consistently determined higher reductions. In addition, flow cytometry results did not show statistically significant differences after exposure to both Adper™ Scotchbond™ 1 XT and Scotchbond™ Universal. However, the exposure to Clearfil™ SE Bond 2 determined significant changes in the culture’s cell cycle. Although the analysis of the types of cell death shown around 70% of live cells when exposing the cultures to Clearfil™ SE Bond 2 at both 25% and 50% concentration, these cells are not metabolic active and changes on the cell cycle are noticeable. The cell cycle alterations corroborate that, even in the surviving cells, cytotoxicity is observed with the blockage of cell cycle progression in G0/G1. This way, we can predict that exposure to adhesives may not immediately lead to cell death but reduces cell’s functions and the proliferative capacity of the cultures. These results support that of the three tested adhesives, Clearfil™ SE Bond 2 was the most cytotoxic.

On the contrary, in studies using dentin barrier models, etch-and-rinse adhesives are shown to be the most cytotoxic [24,27,38]. This can be explained by in vivo studies focusing on the diffusion of resins into the dentinal tubules towards the pulp [39,40]. The Adper™ Scotchbond™ 1 XT adhesive system requires the acid etching of the dental substrate before application. The dentin etching can be deleterious for the collagen fibers, proportional to the concentration and application time of the phosphoric acid [22]. In addition, the exposure of dentinal tubules may favor the progression of the adhesive solution towards the pulp [12]. Scotchbond™ Universal adhesive system can be applied over etched dentin, and in this case, the same concerns regarding adhesive diffusion into the tubules are present. In both cases, we can assume diffusion facilitated in etched dentin and the adhesives cytotoxicity to be higher. However, despite the present results on Clearfil™ SE Bond 2 cytotoxicity, this system is clinically applied over non-etched dentin. Incorporating the smear layer into the adhesive interface constitutes an additional barrier that prevents the adhesive from infiltrating deeply in the direction of the pulp [18]. Additionally, the degree of conversion of the simplified adhesive systems seems to be inferior compared with the conventional presentations [28]. These results may be clinically significant since, after pulp damage, cell signaling events result in reparative dentin or a calcified bridge formation. Additionally, the death of cells directly exposed to adhesives or the acid at etching were reported. Nevertheless, our results do not point to simplified adhesives to be more cytotoxic. Further in vitro studies are necessary to clarify the adhesives dentinal diffusion, using simulated pulpal pressure so that the cytotoxicity of these materials can be assessed in conditions closer to the clinical reality.

The present study is innovative by simultaneously comparing adhesives from the three adhesive strategies using the same experimental conditions. We observed a cell viability reduction and cytotoxicity dependent on the adhesive type, where Clearfil™ SE Bond 2 was the most cytotoxic for the cultures. Studies assessing the types of cell death and cell cycle are scarce. Our study shows that adhesives may interfere with cell cycle, mostly by reducing the number of cells with capacity for proliferation. Additionally, we found the assessment at different incubation periods relevant to evaluating the culture’s behavior when in contact with these materials. The cultures revealed increasing toxicity within more extended incubation periods. The reactivity of extracts is rarely assessed, but it is suggested by the ISO 10993-5, being an additional test. Once again, the self-etch adhesive was revealed to be the most cytotoxic with the more significant inhibition of cultures growth. In future studies, it would be interesting to access if recovery occurs when the cells are no longer exposed to the materials.

Despite significant results being obtained, it is necessary to acknowledge the limitations of the present work regarding a possible clinical translation. In vitro designs do not replicate the complex dynamic of the systems like in vivo studies. This way, materials which show in vitro toxicity are not necessarily toxic when clinically applied in patients [41]. Additionally, when adhesives are clinically applied, cells are exposed for a relatively short period, which is inferior to the longer test periods we used in this study. However, if the adhesive diffuses deeper in the dentinal tubules and cannot be fully polymerized, longer bioavailability will occur, with its cytotoxic consequences. In any case, the obtained information is relevant and can have clinical significance, since when selecting the adhesive system and protocol, it is recommended to consider the dentition, the dental tissues present, the depth of the cavity, and the proximity to the pulp. Acid-etching tooth structure will favor adhesive diffusion and pulp proximity, increasing the possibility for pulp damage. A proper polymerization and handling of these materials are of critical importance to reduce pulp cytotoxicity. Clinical application of adhesive systems in deep caries lesions must be cautious.

## 5. Conclusions

The present study confirmed the in vitro cytotoxicity of Adper™ Scotchbond™ 1 XT, Clearfil™ SE Bond 2, and Scotchbond™ Universal, applied as extracts over cultures of MDPC-23 cells. This cytotoxicity was shown to be dependent on the extract’s concentration and the incubation time.

Although the studied conditions differ from the clinical reality, its use must be careful in deep dentin lesions. Deciduous teeth or young permanent dentition, presence of width pulp chambers, and in the presence of larger dentinal tubules, diffusion of dental adhesives to the pulp may occur and induce cytotoxicity.

## Figures and Tables

**Table 1:** Adhesive strategy, presentation, and clinical steps of adhesive systems application.

Adhesive Strategy	Presentation	Clinical Steps		
		1º Step	2º Step	3º Step
Etch-and-Rinse	3 steps	Acid-etching (enamel and dentin)	Primer	Bond
	2 steps	Acid-etching (enamel and dentin)	One-bottle (primer + bond)	
Self-etch	2 steps	Optional (selective enamel etching)	Primer	Bond
	1 step	Optional (selective enamel etching)	One-bottle (primer + bond)	
Universal	1 step	Optional (selective enamel etching)	One-bottle (primer + bond)	

**Table 2:** Adhesive systems at study, the adhesive strategy, batch number, and presentation.

Adhesive System	Adhesive Strategy	Batch Nr.	Presentation
Adper^™^ Scotchbond^™^ 1 XT (SB1)	Etch-and-Rinse	N952010	One bottle
Clearfil^™^ SE Bond 2 (CSE)	Self-etch	AS0037	Two bottles (primer + bond)
Scotchbond^™^ Universal (SBU)	Universal	80514A	One bottle

**Table 3:** Qualitative cytotoxicity assessment of the cell cultures submitted to the adhesive extracts.

Experimental Condition		Grade
Control		0 (none)
Adper^™^ Scotchbond^™^ 1 XT	25%	1 (slight)
	50%	2 (mild)
Clearfil^™^ SE Bond 2	25%	3 (moderate)
	50%	4 (severe)
Scotchbond^™^ Universal	25%	2 (mild)
	50%	2 (mild)

## Data Availability

Data is contained within the article.
